# Chronic poppers maculopathy: Case report and literature comparison of chronic and acute use

**DOI:** 10.1097/MD.0000000000044079

**Published:** 2025-09-19

**Authors:** Francesco Cappellani, Caterina Gagliano, Jose S. Pulido

**Affiliations:** a Wills Eye Hospital, Thomas Jefferson University, Philadelphia, PA; b Department of Ophthalmology, University of Catania, Catania, Italy; c Faculty of Medicine and Surgery, University of Enna Kore, Enna, Italy; d Ocular Immunology and Rare Diseases Unit, San Marco Hospital, Catania, Italy.

**Keywords:** alkyl nitrite, maculopathy, poppers, poppers maculopathy

## Abstract

**Rationale:**

Alkyl nitrites (“poppers”) are recreational substances known for their vasodilatory and euphoric effects. Increasing evidence links them to a vision-threatening condition termed poppers maculopathy, characterized by foveal yellow spots and outer retinal changes on optical coherence tomography. While most reports describe acute exposure, little is known about long-term consequences. This case describes one of the longest documented durations of chronic poppers use, combined with a literature review to contextualize the clinical spectrum.

**Patient concerns:**

A 68-year-old man with human immunodeficiency virus, type 2 diabetes, hypertension, and depression presented with progressive central vision loss in both eyes persisting for at least 5 years.

**Diagnoses:**

Ophthalmologic examination revealed bilateral foveal yellow spots and retinal pigment epithelium changes. Optical coherence tomography showed photoreceptor and retinal pigment epithelium disruption in the foveal region, more severe in the right eye. Findings were consistent with poppers maculopathy.

**Interventions:**

The patient was counseled regarding the suspected link between his chronic poppers use (≈30 years) and vision loss and advised to discontinue use.

**Outcomes:**

The patient did not return for follow-up, and long-term outcomes remain unknown. Literature review (59 cases, 19 studies) indicated that chronic users present with more severe initial visual impairment compared with acute cases, although many showed partial recovery following cessation.

**Lessons:**

This case underscores the importance of obtaining detailed drug use history in patients with unexplained foveal changes. Chronic exposure to poppers may cause cumulative retinal damage, leading to worse visual outcomes than acute use. Heightened clinical awareness and patient education are essential for the prevention and early recognition of this underdiagnosed condition.

## 1. Introduction

Alkyl nitrites, commonly known as “poppers,” historically used for their euphoric and vasodilatory effects, have been linked to retinal changes. The condition, commonly referred to as poppers maculopathy, is often identified by the presence of bilateral yellow spots at the fovea and specific outer retinal changes observable through optical coherence tomography (OCT). Although the exact mechanism remains unclear, alkyl nitrites are thought to induce retinal damage in poppers maculopathy through nitric oxide-mediated oxidative stress, which may disrupt photoreceptor integrity, particularly in the foveal region.^[1,2]^ This report presents the case of a 68-year-old male with a history of 30 years of chronic poppers use, who exhibited symptoms and diagnostic findings consistent with severe poppers maculopathy, a diagnosis often overlooked in clinical practice. Despite increasing awareness of poppers maculopathy, most published cases focus on acute exposure, with limited data available on the long-term retinal effects of chronic use. This report describes one of the longest documented durations of poppers use in the literature, involving over 3 decades of poppers use, and provides valuable insight into the structural and functional consequences of chronic exposure. Furthermore, we conducted a literature review to evaluate the range of clinical presentations and outcomes associated with acute and chronic exposure to poppers.

## 2. Case presentation

A 68-year-old male patient was referred for evaluation of chronic central vision loss persisting for at least 5 years. His medical history was significant for HIV, diabetes type 2, hypertension, and depression. He was on a regimen of various medications, including antiretrovirals, antidiabetics, and antidepressants. The patient reported gradual, progressive central vision loss over the past 5 years but had not undergone any prior ophthalmologic evaluations, imaging, or treatments.

**Figure 1.**
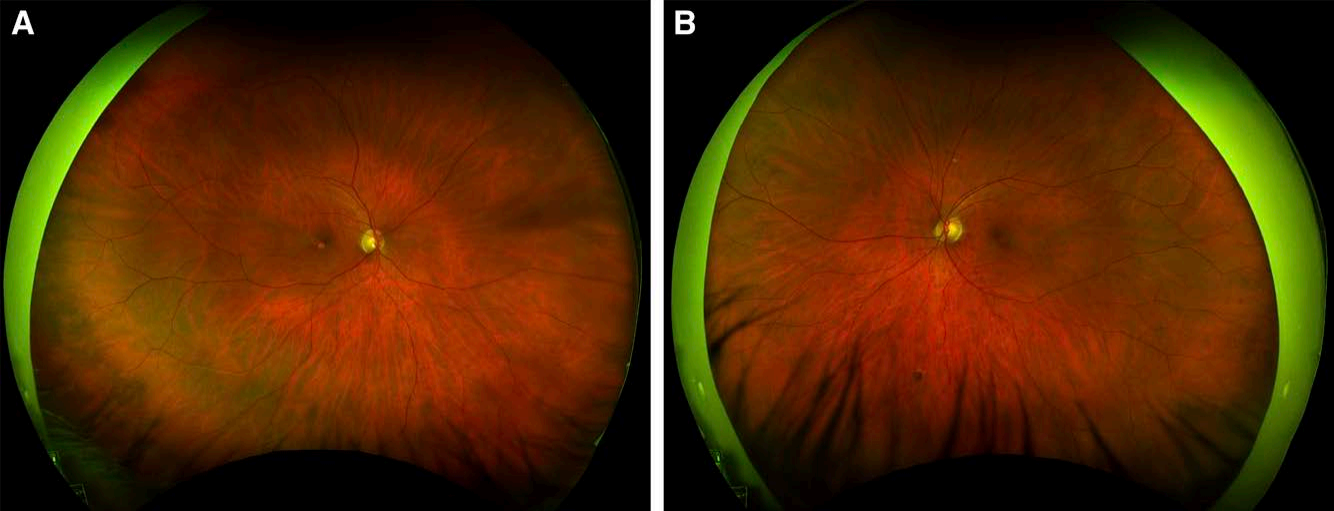
Fundus photographs of the right eye (A) and left eye (B) showing characteristic changes in the fovea consistent with exposure to poppers.

**Figure 2.**
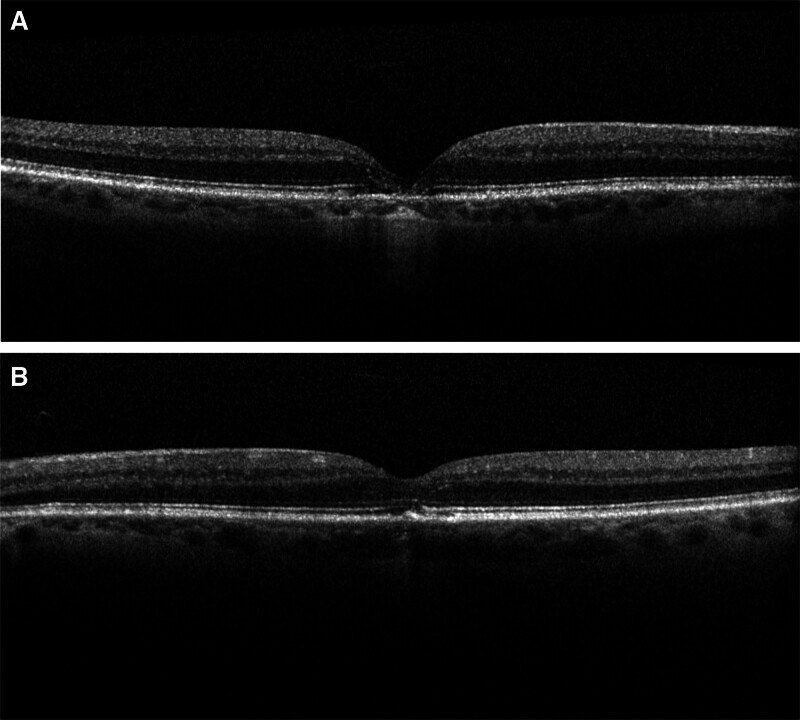
Imaging of both eyes. The right eye (A) shows loss of RPE and photoreceptors in the fovea, while the left eye (B) exhibits milder changes with focal RPE damage. RPE = retinal pigment epithelium.

**Figure 3.**
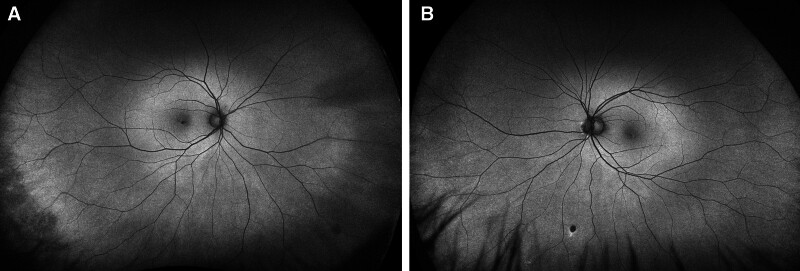
Fundus autofluorescence images of the right eye (A) and left eye (B) showing hypo-autofluorescence in the fovea of both eyes.

**Figure 4.**
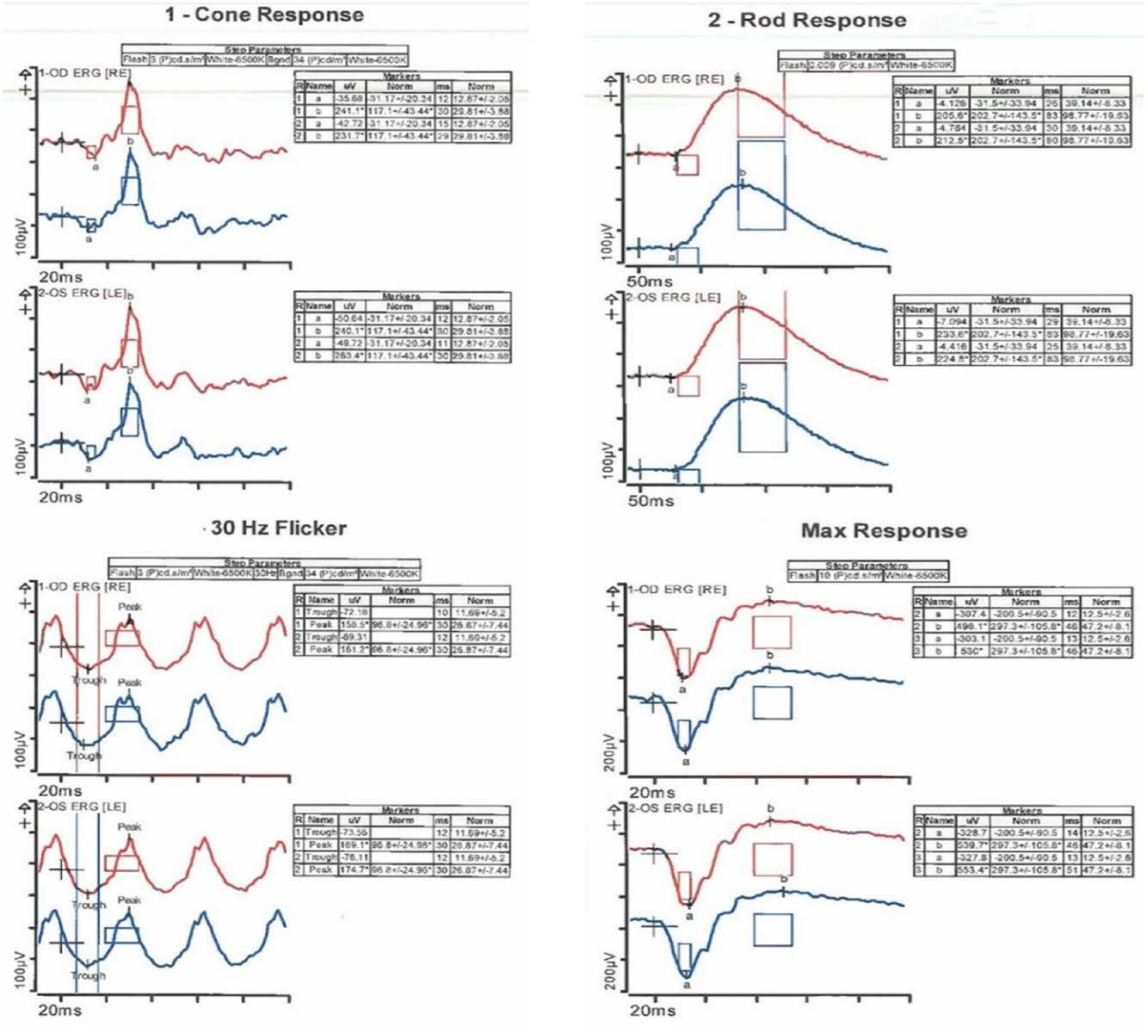
ERG showing rod and cone responses within normal limits. ERG = electroretinogram.

The patient disclosed a 30-year history of recreational poppers use for over a decade daily and more recently 2 to 3 times per week. He denied any history of laser exposure. Ophthalmologic examination revealed a vision acuity of 20/100 in the right eye and 20/60 in the left eye. The anterior segment examination was unremarkable. Intraocular pressure measured 10 mm Hg in both eyes. Extraocular movements and confrontational visual fields were full, and color vision was within normal limits. Dilated fundus examination revealed a yellow spot in the fovea in both eyes with retinal pigment epithelium (RPE) changes, consistent with poppers maculopathy (Fig. 1). OCT of the right eye showed loss of RPE and photoreceptors in the fovea, with the left eye demonstrating similar yet milder changes and focal RPE damage (Fig. 2). Fundus autofluorescence showed hypo-autofluorescence in the fovea of both eyes, and the electroretinogram was within normal limits (Figs. 3 and 4) . The patient was counseled on the suspected link between poppers use and his visual symptoms and was strongly advised to discontinue use. The patient did not return for follow-up, and long-term visual outcomes remain unknown. Other potential causes, including HIV retinopathy, diabetic maculopathy, and medication-related toxicity, were considered unlikely based on the absence of characteristic fundus or OCT findings. The patient has provided informed consent for the publication of this case.

## 3. Methods

### 3.1. Literature review process

A literature search was conducted on PubMed with specific keywords: “poppers maculopathy,” “poppers retinopathy,” “alkyl nitrites maculopathy,” and “alkyl nitrites retinopathy.” We focused on case reports and case series that provided individual patient descriptions, facilitating a detailed analysis. We excluded studies that reported only aggregated data without individual patient details, as these did not allow for stratification by exposure duration (acute vs chronic) or correlation analysis between initial and follow-up Best Corrected Visual Acuity (BCVA). Articles not in English language were also excluded. This strategy yielded 59 cases from 19 studies.^[1–19]^

### 3.2. Data collection

For each case identified through the literature review, we collected data on age, sex, duration of poppers use, reported OCT outcomes, BCVA at presentation, BCVA at follow-up, reported BCVA outcomes, and follow-up duration, noting where specific details were not reported. All BCVA measurements were standardized to the Logarithm of the Minimum Angle of Resolution scale for uniformity and ease of analysis. Patients were categorized into 3 groups based on the duration of poppers use: acute, chronic, and unspecified. For the purposes of this analysis, acute cases were defined as instances involving a single reported use of poppers. Chronic cases included patients who reported using poppers for a duration of 3 or more months. Additionally, an unspecified group was established for cases where the exact duration of poppers use was not reported or was unclear. Data were organized and managed using Microsoft Excel 2021 Version (Microsoft Corporation, Redmond).

### 3.3. Ethical considerations

Ethics committee approval was not required for this study, as it reports a single anonymized case and a literature review of previously published data. Informed consent was obtained from the patient for publication of the case details and images.

### 3.4. Statistical analysis

All statistical analyses were conducted within Microsoft Excel 2021 Version (Microsoft Corporation, Redmond). For the patients identified, we conducted a statistical analysis to examine various aspects, including BCVA at presentation, age distribution, gender distribution, the relationship between initial and final vision, and the relationship between the duration of poppers use and BCVA outcomes. Statistical analysis of variables was tested using the Pearson correlation test to assess the relationship between initial and final BCVA at presentation, averaged for both eyes, considering only the acute and chronic groups, and independent samples *t* test to evaluate differences in BCVA at presentation between acute and chronic groups. Significance was determined a priori as *P* values of <.05. Given the heterogeneity of case report data in terms of quality, completeness, and definitions, all statistical analyses were considered exploratory and aimed to highlight descriptive trends rather than establish definitive associations.

## 4. Results

### 4.1. Patient distribution and demographics

Our analysis included a total of 59 patients derived from 19 studies, categorized based on their reported duration of popper use into 3 categories: 24 unspecified cases, 13 acute cases, and 22 chronic cases (mean duration of poppers use being 10.51 ± 10.50 years). The overall mean age was 40.3 ± 8.96 years (range 23–64). Patients in the acute group had a mean age of 39.1 ± 6.88 years (range 25–52). The mean age for chronic users was 41.8 ± 9.52 years (range 23–64). The gender distribution across all patients showed a statistically significant predominance of male patients, with 57 males (96.6 %) and 2 females (3.4%). The acute group comprised 11 males and 2 females, and the chronic group consisted of 22 males.

### 4.2. BCVA at presentation and follow-up

The mean BCVA at presentation for all patients was 0.217 (20/32 Snellen) ± 0.257. Acute cases showed a slightly better mean BCVA at presentation, 0.149 (20/28 Snellen) ± 0.174, compared to chronic cases, mean 0.244 (20/35 Snellen) ± 0.180 (*P*-value .03). This suggests that chronic use of poppers might be associated with more severe initial visual impairment.

Follow-up BCVA data was available for 36 patients in total, with an additional 3 acute cases reporting improvement without specific numerical data. Specifically, numerical follow-up data BCVA data was reported for 8 acute cases, 12 chronic cases, and 16 unspecified cases. The mean follow-up duration was 7.6 ± 10.4 months for all cases; for acute cases mean follow-up duration was 5 ± 8.7 months, while for chronic cases, it was 10.3 ± 13.2 months. The mean BCVA at follow-up for all patients was 0.110 (20/25 Snellen) ± 0.279 with a median of 0. The mean BCVA at follow-up for acute cases was 0.016 (20/20 Snellen) ± 0.098, with a median of 0. For the chronic group, the mean follow-up BCVA was 0.096 (20/24 Snellen) ± 0.192. In the unspecified group, the mean follow-up BCVA was 0.148 (20/28 Snellen) ± 0.372.

Including only patients with both initial and final vision reported, a correlation analysis was conducted to assess the relationship between initial and final BCVA across all groups. The correlation coefficient was calculated to be 0.732. This result indicates a positive linear relationship between initial and final vision, suggesting that better initial BCVA is a good predictor of better BCVA at follow-up.

### 4.3. OCT and BCVA outcomes

OCT improvements were reported in 31 cases, with 11 not showing improvement and 17 not available. OCT and BCVA outcomes for each group, alongside mean BCVA values at presentation and follow-up, standard deviations, and medians for each group, are comprehensively reported in Table 1.

**Table 1:** BCVA and OCT outcomes.

Group	Cases	BCVA at presentation mean (SD)	BCVA at presentation median	BCVA at follow-up mean (SD)	BCVA at follow-up median	BCVA improvement (yes/no/NA)	OCT improvement (yes/no/NA)
All cases	59	0.217 (0.257)	0.2	0.110 (0.279)	0	25 yes, 14 no,20 NA	31 yes, 11 no, 17 NA
Acute	13	0.149 (0.174)	0.1	0.016 (0.098)	0	8 yes, 3 no, 2 NA	7 yes, 3 no, 3 NA
Chronic	22	0.244 (0.180)	0.2	0.096 (0.192)	0.1	8 yes, 4 no, 10 NA	10 yes, 4 no, 8 NA
Unspecified	24	0.229 (0.339)	0.2	0.148 (0.372)	0	9 yes, 7 no, 8 NA	14 yes, 4 no, 6 NA

BCVA = Best Corrected Visual Acuity, NA = not available, OCT = optical coherence tomography.

## 5. Discussion

The patient’s long-standing use of poppers and the absence of other significant risk factors or ocular abnormalities pointed toward a diagnosis of chronic poppers maculopathy. Although the patient had systemic conditions known to affect the retina, including HIV and diabetes, there were no clinical or imaging signs of HIV or diabetic retinopathy. The electroretinogram was normal, and no medication known to cause maculopathy was identified in his regimen. The bilateral foveal changes, yellow spots, and OCT findings were consistent with previously described features of poppers maculopathy.

Compared to previously reported cases of chronic poppers use, our patient had one of the longest documented exposure durations (30 years vs a mean of 10.5 years in the chronic group). His visual acuity at presentation (20/100 in the right eye and 20/60 in the left eye) was also worse than the average reported in chronic cases (mean 0.244 Logarithm of the Minimum Angle of Resolution, or approximately 20/35 Snellen).

There is currently no established treatment for poppers maculopathy. However, several case reports have noted partial or complete improvement in visual symptoms and OCT findings following cessation of poppers use. While there is no recognized treatment regimen and the prognosis remains uncertain, the role of abstinence might help.^[14]^

The exact mechanism by which poppers induce maculopathy remains unclear, but it has been hypothesized that it might be similar to photic injury. In this scenario, nitric oxide, a component of poppers, could potentially increase photosensitivity, leading to retinal damage.^[20]^ Additionally, because nitric oxide is a potent vasodilator, acute changes in ocular perfusion pressure might contribute to retinal injury.^[21]^ Further complicating the diagnosis of poppers maculopathy is its clinical similarity to photic injury, particularly in terms of the disruptions observed in the ellipsoid zone on OCT. Crucially, a distinguishing factor between these conditions lies in the patient’s history.

This case report highlights the importance of meticulous and comprehensive patient history, encompassing details of both drug use and light exposure, to accurately differentiate between these conditions. Moreover, the first case of central retinal vein occlusion following poppers use, reported in 2024, significantly broadens the spectrum of ocular risks associated with these substances.^[22]^ This recent development emphasizes the critical need for heightened clinical awareness and patient education regarding the risks of recreational poppers use.

Furthermore, in light of our literature review and statistical analysis, we observe that chronic poppers use is associated with more severe initial presentations of maculopathy, as indicated by the BCVA at presentation. This suggests a cumulative detrimental effect on the retina with prolonged exposure. The follow-up data, despite being limited, presents a trend of visual acuity stabilization or improvement. The positive correlation between initial and final BCVA further implies that initial visual status may predict long-term outcomes. However, the lack of comprehensive follow-up data on a substantial number of cases represents a notable gap in the literature, emphasizing the need for more detailed longitudinal research to better understand the full spectrum of poppers maculopathy.

This study has several limitations. The case report is limited by the absence of prior ophthalmologic records and lack of follow-up, which restricts the ability to assess disease progression or outcomes over time. Additionally, the literature search was limited to PubMed, which may have led to the exclusion of relevant studies indexed in other databases. Moreover, the literature review is based on retrospective case reports and case series, many of which vary in quality, completeness, and duration of follow-up. To enable subgroup analysis and correlation testing, only studies with individual patient-level data were included, this exclusion may have introduced selection bias and limited the comprehensiveness of the review. Finally, the statistical analyses performed should be interpreted as exploratory, given the heterogeneity in case characteristics and the descriptive nature of the underlying data.

## 6. Conclusions

In summary, this case, alongside the increasing reports of poppers maculopathy, calls for enhanced efforts in patient education. It is essential to raise awareness not only among potential users but also within the broader healthcare community about the potential vision-threatening effects of acute and chronic popper maculopathy. Our case highlights the need for a comprehensive patient history, with particular emphasis on specific inquiries about the use of alkyl nitrites, commonly referred to as poppers, particularly when faced with these specific foveal findings and in the absence of other obvious risk factors. Educating patients about the risks, particularly in settings where poppers use is more prevalent, could play a pivotal role in preventing future cases of maculopathy and preserving ocular health.

## Author contributions

**Conceptualization:** Francesco Cappellani, Jose S. Pulido.

**Data curation:** Francesco Cappellani, Caterina Gagliano, Jose S. Pulido.

**Formal analysis:** Francesco Cappellani, Jose S. Pulido.

**Funding acquisition:** Jose S. Pulido.

**Investigation:** Francesco Cappellani, Caterina Gagliano.

**Methodology:** Jose S. Pulido.

**Supervision:** Jose S. Pulido.

**Validation:** Jose S. Pulido.

**Visualization:** Caterina Gagliano.

**Writing – original draft:** Francesco Cappellani.

**Writing – review & editing:** Francesco Cappellani, Caterina Gagliano, Jose S. Pulido.
